# Apolipoprotein A1 deficiency in mice primes bone marrow stem cells for T cell lymphopoiesis

**DOI:** 10.1242/jcs.258901

**Published:** 2021-11-16

**Authors:** Amber B. Ouweneel, Myrthe E. Reiche, Olga S. C. Snip, Robbert Wever, Ezra J. van der Wel, Frank H. Schaftenaar, Soňa Kauerova, Esther Lutgens, Miranda Van Eck, Menno Hoekstra

**Affiliations:** 1 Division of BioTherapeutics, Leiden Academic Centre for Drug Research, Leiden University, 2333CC Leiden, The Netherlands; 2 Department of Medical Biochemistry, Amsterdam Cardiovascular Sciences, Amsterdam University Medical Centers, University of Amsterdam, 1105AZ Amsterdam, The Netherlands; 3 Laboratory for Atherosclerosis Research, Institute for Clinical and Experimental Medicine, 12111 Prague, Czech Republic

**Keywords:** Apolipoprotein, High-density lipoprotein, Bone marrow, Stem cells, Lymphopoiesis

## Abstract

The bone marrow has emerged as a potentially important target in cardiovascular disease as it generates all leukocytes involved in atherogenesis. In the current study, we evaluated whether a change in bone marrow functionality underlies the increased atherosclerosis susceptibility associated with high-density lipoprotein (HDL) deficiency. We found that HDL deficiency in mice due to the genetic lack of hepatocyte-derived apolipoprotein A1 (APOA1) was associated with an increase in the Lin^−^Sca-1^+^Kit^+^ (LSK) bone marrow stem cell population and lymphoid-primed multipotent progenitor numbers, which translated into a higher production and systemic flux of T cell subsets. In accordance with APOA1 deficiency-associated priming of stem cells to increase T lymphocyte production, atherogenic diet-fed low-density lipoprotein receptor knockout mice transplanted with bone marrow from APOA1-knockout mice displayed marked lymphocytosis as compared to wild-type bone marrow recipients. However, atherosclerotic lesion sizes and collagen contents were similar in the two groups of bone marrow recipients. In conclusion, systemic lack of APOA1 primes bone marrow stem cells for T cell lymphopoiesis. Our data provide novel evidence for a regulatory role of HDL in bone marrow functioning in normolipidemic mice.

## Abstract (precis)

**Summary:** Changes in cholesterol metabolism, that is, in high-density lipoprotein levels, can significantly impact leukocyte numbers via modulating bone marrow functionality.

## INTRODUCTION

Atherosclerosis, narrowing of the vessel lumen due to arterial cholesterol deposition in response to chronic hypercholesterolemia, represents the primary cause of cardiovascular pathologies, such as coronary artery disease, myocardial infarction and stroke. Current therapies aimed at reducing pro-atherogenic low-density lipoprotein (LDL)-cholesterol levels only reduce cardiovascular disease risk by 15–25% (Josan et al., 2008). As such, alternative therapeutic approaches need to be developed to overcome the residual atherosclerotic cardiovascular disease incidence.

Importantly, accumulating evidence supports the hypothesis that beneficially impacting the number or activation state of leukocytes may also constitute a valuable therapeutic approach to treat atherosclerotic cardiovascular disease. For example, SahBandar et al. (2020) found that a shift in the circulating monocyte population towards the intermediate/inflammatory subtype, i.e. expressing CD16 and high levels of CD14 at their surface, is correlated with a higher carotid intima-media thickness – a surrogate marker of atherosclerosis burden – in the human general population. In addition, Höpfner et al. (2019) and Rogacev et al. (2012) have shown that absolute blood counts of CD14/CD16 (CD16A and CD16B, collectively CD16, are also known as FCGR3A and FCGR3A, respectively) double-positive monocytes can predict major adverse cardiac events in coronary artery disease patients. Furthermore, mass cytometry analysis of human atherosclerotic lesions by Fernandez et al. (2019) has indicated that both CD4^+^ helper T cells and CD8^+^ (CD8A and CD8B) cytotoxic T cells in plaques exhibit a relatively high and heterogeneous activation state as compared to their counterparts in the blood compartment. In further support, Depuydt et al. (2020) have recently shown that human atherosclerotic lesions are rich in activated T cells that also express multiple granzyme species.

Although in certain pathological states, blood cells can also be generated outside the bone marrow, i.e. through extramedullary hematopoiesis, the great majority of circulating leukocytes is normally derived from hematopoietic progenitors within the bone marrow. In accordance with the notion that the bone marrow compartment itself may represent an interesting therapeutic target in the cardiovascular disease setting, several preclinical observations have suggested that functional changes in leukocyte homeostasis induced at the bone marrow level are able to significantly impact atherosclerosis susceptibility. Defective cholesterol efflux from bone marrow stimulates hematopoietic stem cell proliferation and increases granulocyte-monocyte progenitor (GMP) and common myeloid progenitor (CMP) numbers, which is associated with monocytosis, neutrophilia and eosinophilia, and increased atherosclerosis under hypercholesterolemic conditions (Yvan-Charvet et al., 2007, 2010). Studies by van Kampen et al. (2014) and Seijkens et al. (2014) have shown that the induction of hypercholesterolemia through feeding mice a diet enriched in cholesterol and fat similarly primes the bone marrow to produce monocytes and granulocytes and stimulates atherosclerosis susceptibility.

High-density lipoproteins (HDLs) are considered to be potent anti-atherogenic moieties (Choi et al., 2017). Liver-derived apolipoprotein A1 (APOA1) is the primary protein constituent of HDL particles and, as a result, genetic APOA1 deficiency is associated with isolated HDL deficiency and a high predisposition for the development of atherosclerosis in humans (Schaefer et al., 2010). Importantly, atherogenic diet-fed APOA1×LDL receptor double-knockout (DKO) mice not only display the expected increase in atherosclerosis susceptibility (Moore et al., 2003), but also suffer from chronic inflammation as exemplified by enlarged lymph nodes, splenomegaly and the induction of (fatal) skin disease (Zabalawi et al., 2003; Wilhelm et al., 2009). It thus appears that APOA1 deficiency significantly impacts the immune system.

In the current study, we tested whether the virtual absence of HDL due to a genetic deficiency in hepatocyte-derived APOA1 alters bone marrow functionality and thereby changes the immune status and atherosclerosis susceptibility of mice. Hereto, we (1) investigated the bone marrow phenotype of HDL-deficient APOA1-knockout (KO) mice and normolipidemic wild-type controls, and (2) evaluated the effect of a potential change in bone marrow functionality on atherosclerosis susceptibility through a bone marrow transfer from the two types of mice into hypercholesterolemic LDL receptor KO mice.

## RESULTS

To uncover a potential effect of HDL deficiency on bone marrow stem cell number and functionality, we isolated bone marrow and blood from age-matched male wild-type (WT) and APOA1 KO mice fed a standard chow diet. Plasma cholesterol measurements verified the presence of severe hypocholesterolemia in APOA1 KO mice as a result of the genetic lack of APOA1, with plasma total cholesterol levels of 23±1 mg/dl versus 90±2 mg/dl in WT mice (mean±s.e.m., *P*<0.001).

**Fig. 1.**
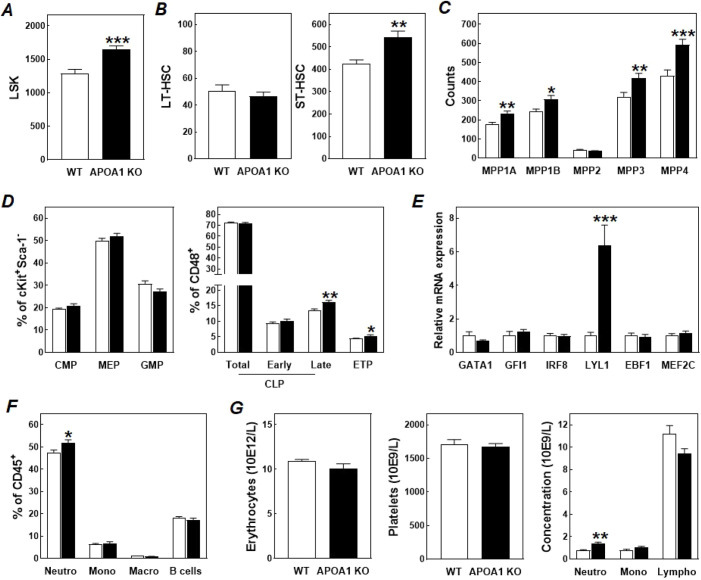
**Total body APOA1 deficiency modulates the bone marrow phenotype and circulating cell count.** Numbers of total (A) and long-term (LT-HSC) and short-term (ST-HSC) hematopoietic stem cells (B), multipotent progenitor subtypes (C), myeloid, megakaryocyte-erythroid, granulocyte-monocyte, lymphoid, and thymocyte progenitor lineages (D), and relative mRNA expression levels of genes involved in hematopoiesis (E), and neutrophil (Neutro), monocyte (Mono), macrophage (Macro), and B cell amounts (F) in bone marrow from wild-type (WT; white bars) and APOA1 knockout mice (APOA1 KO; black bars). (G) Concentrations (units of 10×10^9^ cells/l) of erythrocytes, platelets, neutrophils, monocytes, and lymphocytes in tail blood of wild-type (WT; white bars) and APOA1 knockout mice (APOA1 KO; black bars). Data represent mean±s.e.m. from 16 mice per group. *P<0.05; **P<0.01, ***P<0.001 versus WT (two-tailed unpaired *t*-test).

Flow cytometric analysis on bone marrow single cell suspensions revealed that genetic APOA1 deficiency is associated with a significant increase in the hematopoietic lineage-negative (Lin^−^), stem cell antigen-1-positive (Sca-1^+^; Sca-1 is also known as Ly6A), c-Kit^+^ (LSK) stem cell numbers, both in terms of absolute counts (+29%; *P*<0.001; Fig. 1A) and expressed as fraction of Lin^−^ bone marrow cells (0.87±0.05% for APOA1 KO mice versus 0.67±0.04% for WT mice; mean±s.e.m., *P*<0.01). The increase in total LSK cells is not due to a higher CD150^+^CD48^−^ (CD150 is also known as SLAMF1) long-term hematopoietic stem cell (LT-HSC) count (Fig. 1B), but can rather be attributed to a higher number of short-term CD150^−^ CD48^−^ hematopoietic stem cells (ST-HSC; +18%; *P*<0.01) (Fig. 1B), as also judged by the associated increases in multipotent progenitor (MPP) 1A and 1B levels (Fig. 1C).

The significant increase in MPP3 (+31%; *P*<0.01) and unchanged MPP2 numbers (Fig. 1C) were not associated with a concomitant change in the frequency of common myeloid progenitors (CMPs), megakaryocyte-erythroid progenitors (MEPs) or granulocyte-monocyte progenitors (GMPs) (Fig. 1D). As shown in Fig. 1E, bone marrow relative mRNA expression levels of the erythroid transcription factor GATA1 (Pevny et al., 1995) and the transcription factors growth factor-independent 1 transcription repressor (GFI1) and interferon-regulatory factor 8 (IRF8), which play a crucial role in the generation of neutrophils (Karsunky et al., 2002) and the normal development of monocytes and dendritic cells (Hambleton et al., 2011), were not affected by the absence of APOA1. In agreement, bone marrow CD115^+^ monocytes and F4/80^+^ monocyte-derived macrophages numbers (Fig. 1F) along with blood erythrocyte, platelet and monocyte concentrations (Fig. 1G) were almost identical in APOA1 KO mice and WT controls. Routine hematological analysis did uncover an increase (+73%; *P*<0.01) in blood neutrophil levels in response to APOA1 deficiency (Fig. 1G). However, since only a small increase (+9%; *P*<0.05) in bone marrow Ly6G^+^ neutrophil counts was detected (Fig. 1F), and APOA1 was previously found to directly influence neutrophil migration and activation (Liao et al., 2005; Murphy et al., 2011), it is anticipated that the apparent neutrophilia is not primarily due to an altered bone marrow neutrophil output.

As can be appreciated from Fig. 1C, amongst the different bone marrow MPP populations we detected the most striking increase in the number of lymphoid primed multipotent progenitors (LMPPs, marked by MPP4; +48%; *P*<0.001). This coincided with a marked 6.4-fold increase (*P*<0.001; Fig. 1E) in relative mRNA expression levels of lymphoblastic leukemia 1 (LYL1), a basic-HLH transcription factor that regulates lymphoid specification of multipotent bone marrow progenitors to facilitate thymocyte and T cell development (Zohren et al., 2012). In accordance with the notion that bone marrow stem cells from APOA1 KO mice may generate relatively more thymocyte precursors, we observed a significant increase in terminally differentiated CD48^+^CD127^+^CD27^high^ (CD127 is also known as IL7R) late common lymphoid progenitor (CLP) amounts (+7%; *P*<0.01) and CD48^+^CD127^−^CD27^high^ early thymocyte progenitors (ETPs; +10%; *P*<0.05) as fractions of total CLP (CD48^+^CD127^+^) counts and a similar early (CD48^+^CD127^+^CD135^+^) fraction (CD135 is also known as FLT3) (Fig. 1D). In contrast to the apparent stimulation of bone marrow T lineage differentiation, no difference was seen in the relative mRNA expression levels of the B lineage specification-driving transcription factors early B cell factor 1 (EBF1; Lin and Grosschedl, 1995) and myocyte enhancer factor 2C (MEF2C; Stehling-Sun et al., 2009) or the actual number of CD19^+^ B cells residing in the bone marrow (Fig. 1G).

**Fig. 2.**
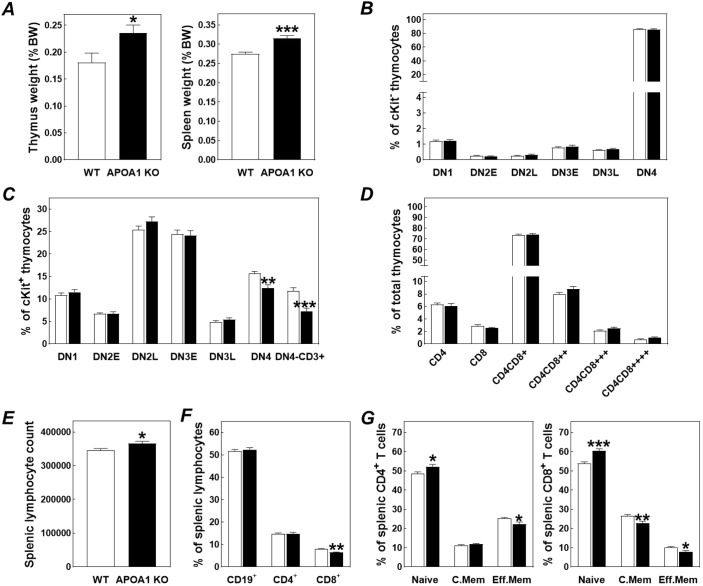
**Total body APOA1 deficiency is associated with enlargement of the thymus and spleen.** Relative thymus and spleen weights (A), thymic CD4 and CD8 double-negative (DN) thymocyte (B,C) and double-positive thymocyte and singe-positive T cell (D) fractions, total splenocyte numbers (E), relative lymphocyte fractions within the spleen (F), and the distribution of naive, central memory, and effector memory subtypes over splenic CD4^+^ and CD8^+^ T cell populations (G) in wild-type (WT; white bars) and APOA1 knockout mice (APOA1 KO; black bars). Data represent mean+s.e.m. from 16 mice per group. *P<0.05; **P<0.01, ***P<0.001 versus WT (two-tailed unpaired *t*-test).

As it appeared that total body APOA1 deficiency is probably associated with enhanced production and migration of T cell progenitors from the bone marrow compartment, we subsequently focused on the potential concomitant effect on the morphology and cellular composition of the thymus where T cell progenitor maturation takes place. Body weight-corrected thymus weights were 30% higher (*P*<0.05; Fig. 2A) in APOA1 KO mice as compared to WT controls, which implies that APOA1 deficiency is associated with a higher total number of thymocytes. No genotype-associated difference was found in the thymic relative number of CD4 and CD8 double-negative (DN) c-Kit^−^ precursors that are generated locally within the thymus (Fig. 2B). However, an APOA1 deficiency-associated significant decrease was observed in the DN4 subfraction (−20%; *P*<0.01) within the bone marrow-derived c-Kit^+^ thymocyte population (Fig. 2C). Particularly the c-Kit^+^ DN4 subfraction that highly expressed the general T cell marker CD3 was reduced (DN-CD3^+^; −39%; *P*<0.001; Fig. 2C). However, relative amounts of double-positive thymocytes or mature CD4 and CD8 single-positive T cells within the thymus were not different between the two types of mice (Fig. 2D).

The alterations in thymus weight and cellular composition did not translate into a change in the circulating lymphocyte counts (Fig. 1F). However, the spleen weights, corrected for body weight, were significantly higher in APOA1 KO mice as compared to WT mice (+15%; *P*<0.001; Fig. 2A). In concordance with this, flow cytometric analysis revealed an increase in total lymphocyte counts (Fig. 2E), while the relative contribution of CD19^+^ B cells and CD4^+^ T cells to the total number of splenocytes was not significantly different between the two genotypes (Fig. 2F). In contrast, the CD8^+^ T cell fraction was reduced in APOA1 KO spleens (−18%; *P*<0.001; Fig. 2F). A significant shift in T cell polarization status towards the newly acquired, naive (CD62L^+^CD44^−^; CD62L is also known as SELL) subtype both in the CD4^+^ and CD8^+^ splenocyte populations was observed, while the relative numbers of central memory (CD62L^+^CD44^+^), and effector memory (CD62L^−^CD44^+^) T cell populations tended to decrease or were significantly reduced (Fig. 2G). These findings suggest that size of the APOA1 KO spleens was probably increased as a result of an enhanced influx and storage of T cells from the thymus.

**Fig. 3.**
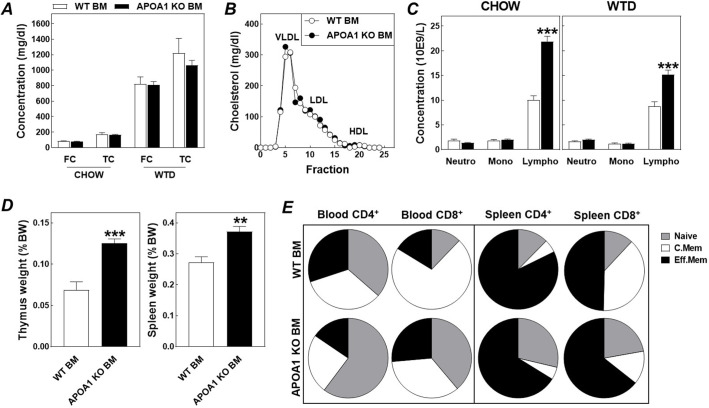
**Repopulation of hypercholesterolemic LDL receptor KO mice with bone marrow from APOA1 KO mice induces lymphocytosis without a parallel change in plasma lipid levels.** Plasma free (FC) and total cholesterol (TC) levels (A), the distribution of cholesterol over the different lipoprotein fractions (B), concentrations of neutrophils (Neutro), monocytes (Mono), and lymphocytes (Lympho) in tail blood (C), relative thymus and spleen weights (D), and the distribution of naive, central memory (C.Mem), and effector memory (Eff.Mem) subtypes over CD4^+^ and CD8^+^ T cells in the blood and spleen (E) in LDL receptor KO mice transplanted with wild-type bone marrow (WT BM; *n*=8) or APOA1 KO bone marrow (APOA1 KO BM; *n*=16). Chow, chow diet feeding conditions; WTD; Western-type diet feeding conditions; VLDL, very-low-density lipoprotein; LDL, low-density lipoprotein; HDL, high-density lipoprotein. Data represent mean+s.e.m. *P<0.05; **P<0.01, ***P<0.001 versus WT BM (two-tailed unpaired *t*-test).

To verify the potential effect of the APOA1/HDL deficiency-associated changes in bone marrow functionality on atherosclerosis susceptibility, we transplanted bone marrow from APOA1 KO mice and WT controls into atherosclerosis-prone, hypercholesterolemic LDL receptor KO mice. LDL receptor KO recipient mice were maintained on a regular chow (non-atherogenic) low fat diet for 8 weeks to enable full repopulation of all bone marrow-derived cell populations in the blood compartment, before the subsequent challenge with a high cholesterol/high fat Western-type diet to stimulate atherosclerotic lesion development. The difference in donor bone marrow phenotype did not influence plasma free or total cholesterol levels under chow diet feeding conditions (Fig. 3A). However, routine hematological analysis revealed the anticipated increase (+118%; *P*<0.001) in blood lymphocyte counts in chow diet-fed APOA1 KO bone marrow recipients as compared to chow diet-fed LDL receptor knockout mice that had received WT bone marrow (Fig. 3C). No major change in blood monocyte or neutrophil concentrations was detected (Fig. 3C). The effect of HDL deficiency on donor bone marrow functionality (i.e. priming of stem cells to produce lymphocytes), was thus clearly maintained after transplantation into the LDL receptor KO mice with normal APOA1 levels.

The blood phenotype was essentially preserved upon the 6-week atherogenic Western-type diet challenge. APOA1 KO bone marrow recipients still exhibited marked lymphocytosis (+73%; *P*<0.01) (Fig. 3C). In addition, plasma free cholesterol and total cholesterol levels were increased to a similar extent by the atherogenic diet feeding in the two bone marrow recipient groups (Fig. 3A). Furthermore, the distribution of the plasma cholesterol pool over the different lipoprotein fractions at sacrifice was not affected by the bone marrow genotype (Fig. 3B). Notably, as can be appreciated from Fig. 3D, the APOA1 KO bone marrow genotype-associated lymphocytosis was paralleled by significantly higher body weight-corrected weights of the thymus (+83%; *P*<0.001) and the spleen (+37%; *P*<0.001). In further support of the hypothesis that the APOA1 KO bone marrow genotype was also associated with an enhanced generation of new T lymphocytes under the hypercholesterolemic conditions, analysis of both the circulating and splenic T cell populations revealed the anticipated shift towards the naive subtype in Western-type diet-fed APOA1 KO bone marrow recipients as compared to WT bone marrow recipient controls (Fig. 3E). As a result, naive CD4^+^ and CD8^+^ T cell fractions in the blood compartment increased from respectively 1.1±0.3% and 0.8±0.2% in WT bone marrow recipients to 3.6±0.8% and 1.7±0.3% in APOA1 KO bone marrow recipients (mean±s.e.m.; *P*<0.05 for both).

**Fig. 4.**
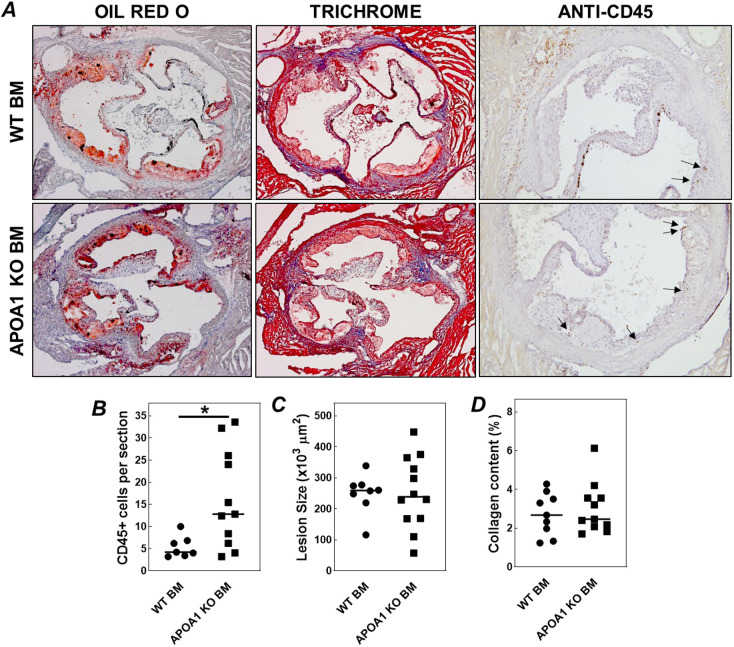
**Repopulation of hypercholesterolemic LDL receptor KO mice with bone marrow from APOA1 KO mice does not change the atherosclerosis susceptibility.** (A) Representative images of aortic root atherosclerotic lesions stained for neutral lipids with Oil Red O (left), Trichrome to identify collagen in blue (middle), and anti-CD45 antibody to identify T cells (right). Quantifications of the number of T cell per section (B), total atherosclerotic lesion sizes (C) and relative lesional collagen contents (D) in Western-type diet-fed LDL receptor KO mice transplanted with wild-type bone marrow (WT BM; circles) or APOA1 KO bone marrow (APOA1 KO BM; squares). Arrows in A point towards CD45^+^ cells. Data points in B–D represent individual mice, while horizontal lines indicate the respective group averages. *P<0.05 versus WT BM (two-tailed unpaired *t*-test).

To establish whether the systemic accumulation of naive T cells into LDL receptor KO mice in response to transplantation with bone from HDL deficient APOA1 KO mice affects atherosclerotic plaque development, the atherosclerosis extent was measured in the aortic root after 6 weeks of Western-type diet feeding. As evident from the representative images in Fig. 4A, early-stage atherosclerotic lesion formation was observed in both experimental groups that did also contain some CD45^high^ cells (CD45 is also known as PTPRC), i.e. T cells. In line with the higher blood T cell counts, donor APOA1 deficiency was also associated with an increase in the number of CD45^+^ cells T cells in atherosclerotic lesions (+200%; *P*<0.05; Fig. 4B). However, atherosclerotic plaques from APOA1 KO bone marrow transplanted mice were of a similar size as plaques from WT bone marrow transplanted mice (252±33×10^6^ µm^2^ versus 249±22×10^6^ µm^2^, mean±s.e.m.; Fig. 4C). Masson's Trichrome staining verified that collagen contents of the plaques were also similarly low in both groups of bone marrow recipients, as expected for early lesions (2.5±0.4% for APOA1 KO bone marrow versus 3.0±0.4% for WT bone marrow; Fig. 4D). In conclusion, enhanced T cell lymphopoiesis and the associated T cell accumulation in plaques of APOA1 KO bone marrow recipients is not associated with a concomitant change in high cholesterol/high fat diet-induced atherosclerosis susceptibility.

## DISCUSSION

In the current study, we have found that HDL deficiency due to a systemic lack of hepatocyte-derived APOA1 in mice is associated with priming of hematopoietic stem cells to generate early thymic progenitors that efficiently (re-)populate the thymus and subsequently produce naive T lymphocytes in total body APOA1 KO mice as well as after transplantation of bone marrow into irradiated hypercholesterolemic LDL receptor KO mice.

The relatively high blood lymphocyte concentrations observed in LDL receptor KO mice after transplantation with bone marrow from APOA1 KO mice did not constitute a change in atherosclerosis susceptibility. As such, in contrast to our original hypothesis, we anticipate that changes in the bone marrow functionality do not play a significant role in the increased atherosclerosis susceptibility detected in HDL deficient human APOA1 mutation carriers and APOA1×LDL receptor DKO mice. However, our finding that a specific naive T cell accumulation in APOA1 KO bone marrow recipients does not increase atherosclerotic cardiovascular disease risk is not surprising. Olson et al. (2020) previously showed that there was no statistically significant association between CD4^+^ naive T helper cell blood counts and angina or myocardial infarction incidence in two independent human patient cohorts. It rather appears that the activity of effector T cells is decisive for the aggravation of atherosclerotic lesion development. In line with this, Betjes et al. (2010) showed that a shift towards the pro-inflammatory CD28^null^ subclass within the total circulating CD4^+^ helper T cell population was associated with a higher prevalence of atherosclerotic cardiovascular disease.

The question remains as to what the regulatory mechanism is behind the APOA1 deficiency associated priming of hematopoietic stem cells to generate early thymic progenitors that induce thymus hyperplasia after bone marrow transplantation. In this context it is important to acknowledge that we have also repeatedly detected lymphocytosis in atherogenic diet-fed LDL receptor KO mice that were transplanted with bone marrow from hypocholesterolemic (HDL deficient) mice genetically lacking the ATP-binding cassette transporter A1 (ABCA1) (van Eck et al., 2002; Ouweneel et al., 2021). Since ABCA1 KO mice are able to produce functional APOA1 proteins, the impact of genetic APOA1 deficiency on bone marrow T cell lymphopoiesis can therefore likely be attributed to the associated HDL deficiency rather than to the genetic lack of APOA1. HDL deficiency in ABCA1×ABCG1 DKO mice is, however, associated with augmented monocyte and granulocyte, but not accelerated lymphocyte formation (Yvan-Charvet et al., 2010; Westerterp et al., 2012). Importantly, ABCA1×ABCG1 DKO mice are not only HDL deficient, but also already spontaneously accumulate macrophage foam cells in the liver, spleen and lymph nodes due to a major defect in cellular cholesterol efflux (Out et al., 2008). HDL-deficient ABCA1 KO mice do not display this extreme foam cell susceptibility, given that ABCG1 is still able to efflux cholesterol (Out et al., 2008). HDL deficiency in APOA1 KO mice also does not translate into an elevated foam cell susceptibility, which can be explained by the fact that other apolipoproteins, such as, for instance, APOE, may serve as an alternative cholesterol acceptors (Filou et al., 2016). When taking these combined findings into account, it appears that lipid loading primes stem cells for monocyte/granulocyte formation and that isolated HDL deficiency is instead associated with enhanced T cell lymphopoiesis.

In normolipidemic mice, HDL particles are functionally relevant in supplying cholesterol to the adrenals for the production of glucocorticoid species (i.e. corticosterone). HDL-deficient mice therefore generally suffer from glucocorticoid insufficiency (Plump et al., 1996; Hoekstra et al., 2010, 2013). Importantly, Sacedón et al. (1999) have previously shown that depletion of plasma glucocorticoids in rat fetuses through maternal bilateral adrenalectomy is associated with an accelerated maturation and thymic recolonization potential of fetal liver-derived T cell progenitors. It can thus be hypothesized that a diminished glucocorticoid function may dictate the global effect of genetic APOA1 deficiency on the bone marrow progenitors found in our donor mice. In further support of this hypothesis, we have found that (1) isolated HDL deficiency due to genetic lack of APOA1 is not associated with glucocorticoid insufficiency in LDL receptor KO mice given that adrenals likely utilize LDL and very low-density lipoprotein (VLDL) particles as cholesterol sources under hypercholesterolemic conditions and that, probably as a result, (2) LDLR KO mice transplanted with bone marrow from HDL deficient ABCA1×LDL receptor DKO mice do not exhibit lymphocytosis (Hoekstra and Van Eck, 2016; Ouweneel et al., 2021). As such, it will be interesting to study whether disruption of the adrenal glucocorticoid function in mice, i.e. through adrenalectomy, is associated with a similar change in the bone marrow progenitor phenotype as observed in our current HDL deficiency setting.

In conclusion, we have shown that HDL deficiency due to a systemic lack of hepatocyte-derived APOA1 in mice is associated with priming of bone marrow stem cells to produce T lymphocytes, but that the resulting increase in blood lymphocyte concentrations after transplantation of bone marrow from APOA1 KO mice into hypercholesterolemic LDL receptor KO mice does not affect the susceptibility for development of atherosclerotic lesions. Our data provide new evidence for a regulatory role of HDL in bone marrow functioning in normolipidemic mice. Importantly, reduced plasma HDL-cholesterol levels is a common feature in acute and chronic lymphatic leukemia patients (Yavasoglu et al., 2017; Morel et al., 2017; Komiya et al., 2018). Although it is generally assumed that the hypocholesterolemia associated with leukemia results from an overactive uptake and usage of circulating cholesterol by cancer cells, from our novel findings it can be suggested that HDL deficiency rather contributes to the leukemia-related lymphocytosis development in these patients. In light of the interesting observations by Komiya et al. and Yun et al. that low HDL-cholesterol levels are a marker of poorer clinical outcome in patients with lymphocytic leukemia (Komiya et al., 2018; Yun et al., 2021), HDL and its key protein component APOA1 could possibly also serve as therapeutic moieties for the treatment of leukemia.

## MATERIALS AND METHODS

### Mice

WT, APOA1 KO, and LDL receptor KO mice on a C57BL/6 background were bred in house. All experimental mice were group-housed in filter top cages within climate-controlled rooms at the animal research facility of the Gorlaeus laboratories, Leiden, The Netherlands. Animal experiments were performed in accordance with the ARRIVE guidelines and approved by the Dutch Central Commission for Animal experimentation (Centrale Commissie voor Dierproeven) according to the Dutch Law on laboratory animal experimentation and the EU Directive 2010/63/EU. Power calculations were executed to determine the minimal number of mice needed to ensure adequate power to detect a pre-specified effect size using an online calculation tool (https://www.stat.ubc.ca/~rollin/stats/ssize/n2.html).

To investigate the effect of total body APOA1 deficiency, we pooled data from analysis of two separate groups of chow diet-fed 14- to 18-week-old age-matched male WT mice (*n*=6 and *n*=10; total *n*=16) and APOA1 KO mice (*n*=6 and *n*=10; total *n*=16) that were derived through intercrossing of heterozygous APOA1 deficient mice. Mice were anesthetized with a mix of xylazine (70 mg/kg body weight), ketamine (350 mg/kg body weight), and atropine (1.8 mg/kg body weight) and killed by orbital exsanguination, after which the spleen, thymus, and femurs and tibias were collected for further analysis.

To determine the effect of bone marrow APOA1 deficiency on atherosclerosis outcome, two groups of age- and weight-matched 11- to 22-week-old female LDL receptor KO mice were subjected to 2×4.5 Gy total body radiation using a 225 Smart Röntgen source (YXLON international, Copenhagen, Denmark). One day after the irradiation, the LDL receptor KO mice received an injection into the tail vein with 200 µl of a single cell suspension of pooled bone marrow from two male WT mice (WT BM; *n*=8) or two male APOA1 KO mice (APOA1 KO BM; *n*=12), obtained through flushing femurs and tibias with PBS. The drinking water of bone marrow recipient mice was supplemented with antibiotics (83 mg/l ciprofloxacin and 67 mg/l polymixin B sulfate) and 6.5 g/l sucrose starting 7 days prior to transplantation up to 4 weeks after transplantation. After transplantation, mice were fed a regular chow diet during recovery for 11 weeks, after which blood was drawn from the tail vein for hematological and flow cytometric analysis and measurement of the plasma cholesterol concentration. Subsequently, mice were fed a Western-type diet containing 0.25% cholesterol and 15% cocoa butter (Special Diet Services, Witham, Essex, UK) for 6 weeks to exacerbate the hypercholesterolemia extent and stimulate the development of atherosclerotic lesions. At 17 weeks post transplantation, after 6 weeks of Western-type diet feeding, mice were anesthetized with a mix of xylazine (70 mg/kg body weight), ketamine (350 mg/kg body weight) and atropine (1.8 mg/kg body weight) and killed by orbital exsanguination and perfusion with PBS, after which the organs, femurs and tibias were collected for further analysis.

### Plasma cholesterol measurements

Blood obtained from tail vein bleeding was collected in EDTA-coated tubes. Plasma was attained by centrifugation (10 min; 6000 r.p.m.) and stored at −20°C until further use. Plasma free and total cholesterol levels were measured by enzymatic colorimetric assays as described by Out et al. (2007). The distribution of cholesterol over the different lipoproteins in plasma was determined by fractionation of 30 μl of pooled plasma using a Superose 6 column (3.2×300 mm, Smart-System; Pharmacia, Uppsala, Sweden). Total cholesterol content of the effluent was determined using the standard colorimetric assay.

### Hematological analysis

Blood obtained from the tail vein was collected in EDTA-coated tubes and diluted 4× in PBS for analysis of blood cell count using an automated XT-2000iV veterinary hematology analyzer (Sysmex Europe GMBH, Norderstedt, Germany).

### Flow cytometry

To obtain a single-cell suspension of bone marrow cells, flushed bone marrow from the femurs and tibias was strained through a 70 µm nylon mesh (Greiner Bio-One, Kremsmünster, Austria) with PBS. Single-cell suspensions of splenocytes and thymocytes were obtained by straining spleen and thymus through a 70 µm nylon mesh (Greiner Bio-One). Blood was obtained from the tail vein and collected in EDTA-coated tubes. Bone marrow suspension, splenocytes and blood was treated with ammonium-chloride-potassium erythrocyte lysing buffer (0.15 M NH_4_Cl, 10 mM NaHCO_3_, 0.1 mM EDTA, pH 7.3) for the appropriate times.

Flow cytometric analysis on tissue samples from WT and APOA1 KO mice was performed at the Department of Medical Biochemistry, Amsterdam University Medical Centers. Bone marrow, spleen and thymic single-cell suspensions were stained and analyzed using the FACS Fortessa (BD Biosciences, San Jose, CA, USA) and FlowJo v10.5.3 software (FlowJo, LLC). The gating strategy for specific cell populations measured in the bone marrow is provided in Table S1.

Flow cytometric analysis on blood and tissue samples obtained from bone marrow recipients was performed at the Division of BioTherapeutics from the Leiden Academic Centre for Drug Research on a Cytoflex S flow cytometer (Beckman Coulter, Brea, California, USA). The acquired data were analyzed using FlowJo software (FlowJo LLC, Ashland OR, USA). Gates were set according to fluorescence minus one controls.

### Gene expression analysis

Total RNA was isolated from bone marrow by standard phenol-chloroform extraction. Equal amounts of RNA were reverse transcribed and, subsequently, real-time quantitative PCR analysis was executed on the cDNA using an ABI Prism 7500 apparatus (Applied Biosystems, Foster City CA, USA) according to the manufacturer's instructions. Ribosomal protein L37 (RPL37), acidic ribosomal phosphoprotein P0 (36B4; also known as RPLP0), and peptidylprolyl isomerase A (PPIA) were used as reference genes for normalization. Primer sequences are available upon request.

### Atherosclerotic lesion analysis

After whole-body perfusion with PBS, hearts were fixed for 4 h in 3.7% formalin (Formal-fixx; Shandon Scientific Ltd, UK). Fixed hearts were embedded in Sakura O.C.T. Compound™ (Sakura Finetek Europe B.V., Alphen aan de Rijn, The Netherlands), for cryosectioning. Serial sections (10 μm; 70 μm interval) of the aortic root were cut using the Leica CM3050S cryostat. Plaque size in the aortic root was determined by staining for neutral lipids using Oil Red O and Mayer's hematoxylin (Sigma-Aldrich, Zwijndrecht, The Netherlands). Corresponding sections were stained for collagen using Masson's Trichrome (Sigma-Aldrich). Since our anti-CD3 antibody-based T cell staining protocol did not work on these sections, the number of T cells present in atherosclerotic plaques was analyzed using an antibody directed against CD45 that – using our staining protocol – specifically identifies leukocytes with a very high surface CD45 expression, i.e. CD45^high^ T cells, and does not detect macrophages in formalin-fixed cryosections of the aortic root (Seijkens et al., 2019). All images were analyzed blinded by computer-aided morphometric analysis using a Leica DM-RE microscope and LeicaQwin software (Leica Ltd, Cambridge, UK).

### Statistical analysis

Statistical analysis was performed using Graphpad Prism software (San Diego CA, USA, http://www.graphpad.com). Outlier detection was performed using a Grubbs’ test. The significance of differences was calculated using a two-tailed unpaired *t*-test or Mann–Whitney U test if not conforming to Gaussian distribution as assessed by D'Agostino-Pearson omnibus normality test. Probability values <0.05 were considered significant.

## Supplementary Material

Supplementary information
