# Supplementary material for: Apolipoprotein A1 deficiency in mice primes bone marrow stem cells for T cell lymphopoiesis
Source: J Cell Sci. 2021 Nov 16;135(5):jcs258901. doi: 10.1242/jcs.258901 (PMC8645231; doi:10.1242/jcs.258901)
Supplement: Supplementary information [file joces-135-258901-s1.pdf]

Table S1. Gating strategy for specific cell populations within the bone marrow

Name	Marker combination
Lin$^-$	CD3e$^-$,Ly-6G$^-$,Ly-6C$^-$,CD11B$^-$,B220$^-$,TER-119$^-$
LSK	Lin$^-$,c-Kit$^+$,Sca-1$^+$
LT-HSC	Lin$^-$,c-Kit$^+$,Sca-1$^+$,CD150$^+$,CD48$^-$
ST-HSC	Lin$^-$,c-Kit$^+$,Sca-1$^+$,CD150$^-$,CD48$^-$
MPP1a	Lin$^-$,c-Kit$^+$,Sca-1$^+$,CD150$^-$,CD48$^-$, CD135$^-$
MPP1a	Lin$^-$,c-Kit$^+$,Sca-1$^+$,CD150$^-$,CD48$^-$, CD135$^+$
MPP2	Lin$^-$,c-Kit$^+$,Sca-1$^+$,CD150$^+$,CD48$^+$
MPP3	Lin$^-$,c-Kit$^+$,Sca-1$^+$,CD150$^-$,CD48$^+$,CD135$^-$
MPP4	Lin$^-$,c-Kit$^+$,Sca-1$^+$,CD150$^-$,CD48$^+$,CD135$^+$
GMP	Lin$^-$,c-Kit$^+$,Sca-1$^-$, CD16/32$^+$, CD34$^+$
CMP	Lin$^-$,c-Kit$^+$,Sca-1$^-$, CD16/32^{Med}, CD34^{Med}
MEP	Lin$^-$,c-Kit$^+$,Sca-1$^-$, CD16/32$^-$, CD34$^-$
CLP	Lin$^-$,c-Kit^{lo},Sca-1$^+$, CD48$^+$, CD135$^-$, CD127$^+$
Early CLP	Lin$^-$,c-Kit^{lo},Sca-1$^+$, CD48$^+$, CD135$^+$, CD127$^+$
Late CLP	Lin$^-$,c-Kit^{lo},Sca-1$^+$, CD48$^+$, CD135$^+$, CD127$^+$, CD27$^+$
ETP	c-Kit$^{lo/med}$, Sca-1$^{-/lo}$,CD127$^-$, CD27$^+$, CD44$^+$
